# Focal electrical stimulation on an alcohol disorder model using magnetic resonance imaging-compatible chronic neural monopolar carbon fiber electrodes

**DOI:** 10.3389/fnins.2022.945594

**Published:** 2022-09-29

**Authors:** Alejandra Lopez-Castro, Diego Angeles-Valdez, Gerardo Rojas-Piloni, Eduardo A. Garza-Villarreal

**Affiliations:** ^1^Instituto de Neurobiología, Universidad Nacional Autónoma de México, Querétaro, Mexico; ^2^Department of Biomedical Sciences of Cells and Systems, Cognitive Neuroscience Center, University Medical Center Groningen, University of Groningen, Groningen, Netherlands

**Keywords:** MRI, electrodes, carbon fiber, DBS, SUD, AUD, alcohol

## Abstract

Neuromodulation interventions, such as Deep Brain Stimulation (DBS) and repeated transcranial magnetic stimulation (rTMS), are proposed as possible new complementary therapies to treat substance use disorders (SUD) such as alcohol use disorder (AUD). It is hypothesized that neuromodulation may induce neural plasticity in the reward and frontostriatal systems *via* electrical field induction, possibly reducing symptoms. Preclinical self-administration rodent models of AUD may help us gain insight into the effects of neuromodulation therapies on different pathology, as well as the neural mechanisms behind the positive effects. DBS, or any type of brain stimulation using intracranial electrodes in rodents, would benefit from the use of magnetic resonance imaging (MRI) to study the longitudinal effects and mechanisms of stimulation as well as novel targets, as it is a non-invasive technique that allows the analysis of structural and functional changes in the brain. To do this, there is a need for MRI-compatible electrodes that allow for MRI acquisition with minimal distortion of the magnetic field. In this protocol, we present a method for the construction and surgery of chronically implantable monopolar carbon electrodes for use in rats. Unlike conventional electrodes, carbon electrodes are resistant to high temperatures, flexible, and generate fewer artifacts in MRI compared to conventional ones. We validated its use by using a focal electrical stimulation high-frequency (20 Hz) protocol that lasted ∼10 sessions. We propose that this technique can also be used for the research of the neurophysiological bases of the neuromodulatory treatment in other preclinical substance use disorders (SUD) models.

## Introduction

Neuromodulation encompasses technologies that apply electrical currents with a variety of parameters, through implanted or non-implanted electrodes, to achieve a functional activation or inhibition of a group of neurons, pathways, or circuits (Krames et al., 2009). The main neuromodulation techniques used in humans are repetitive transcranial magnetic stimulation (rTMS) and deep brain stimulation (DBS) (Klooster et al., 2016). Currently approved by the Food and Drug Administration (FDA), is rTMS therapy for adjunctive treatment for nicotine use disorder, and DBS for essential tremors, dystonia, obsessive-compulsive disorder, and Parkinson’s disease (Edwards et al., 2017). Alas, no consensus exists regarding the mechanisms of action involved in rTMS or DBS (Diana et al., 2017). The effects and mechanisms of neuromodulation in SUDs can be further tested through the use of animal models (Ankeny et al., 2014) which can provide valuable information. For instance, using magnetic resonance imaging (MRI) in longitudinal animal models for SUDs treated with neuromodulation, researchers may be able to find neuroimaging biomarkers (Aydin et al., 2019), effects, and mechanisms related to clinical outcomes, which could be later translated to human studies (Silva and Bock, 2008; Spanagel, 2017). To study focal repeated stimulation in rats, intracranial electrodes are currently a practical solution. However, there are a few major methodological challenges to use intracranial electrodes in longitudinal studies with MRI. Metal electrodes are most commonly used for stimulation in rats, yet are highly susceptible to create susceptibility artifacts (Dunn et al., 2009), which leads to the loss of signal around the region where the electrode is placed, as well as distortion and lower signal-to-noise ratio (SNR) (Redpath, 1998). For DBS studies in rodents, 140 μm diameter braided platinum/iridium wire electrodes (Van De Berg, 2015), 200 μm diameter braided stainless steel wires (Morimoto et al., 2011) and 250 μm braided silver wires have been used for DBS protocols in conjunction with simultaneous MRI and EEG acquisitions. Platinum/iridium and stainless steel electrodes induce a susceptibility artifact of twice the original electrode diameter and silver electrodes do not increase the artifact compared to the electrode diameter, but there is more signal around the longitudinal diameter of the electrode (Dunn et al., 2009; Young et al., 2011; Derksen et al., 2021). Carbon fiber electrodes have been used in neuroscience as an option for recording (Chuapoco et al., 2019; Joshi-Imre et al., 2019) and stimulating brain regions (Gallino et al., 2019). These electrodes have proven to be a better choice for the improvement of SNR with little to none MRI susceptibility artifacts, due to their physical properties like high resistance, light-weight, and low density, composed of a tensile of 900 GPa, with thermal conductivity of 1,000 W/mK and electric conductivity of 106 S/m (Zhao et al., 2019). However, the viability of its use in the treatment of SUDs and in longitudinal MRI studies has not been tested.

Therefore, this work aims to provide a simple and low-cost approach for assembling chronically implantable monopolar carbon electrodes and their use for repetitive focal stimulation in alcohol use disorder (AUD). Also, we proposed it can be used in other models of SUDs.

## Materials and equipment

### Methods

Tables 1, 2 list the materials and doses required to perform the electrode implantation surgery. The study was approved by the Animal Research Committee of the Instituto de Neurobiología at Universidad Nacional Autónoma de México No. 119-A. All surgical, experimental, and maintenance procedures were carried out in accordance with the Reglamento de la Ley General de Salud en Materia de Investigación para la Salud (Health General Law on Health Research Regulation) of the Mexican Health Ministry that follows the “Guide for the care and use of laboratory animals” (Mexicana Norma Oficial, 1999; National Research Council et al., 2011). Also in accordance with the recommendations of the Institute of Laboratory Animal Resources Commission on Life Sciences (Guide for the Care and Use of Laboratory Animals, 1996) and the Directive 2010/63/EU of the European Parliament and of the Council.

**TABLE 1:** Materials.

1. Lab standard stereotaxic instrument 2. Heat pad (Beurer^®^ HK55) 3. Gas anesthesia mask compatible with the stereotaxic setup. 4. Anesthetic gas vaporizer 5. Isoflurane (SOFLORAN VET PiSA^®^) 6. Anesthesia induction chamber 7. Oxygen supply 8. Surgical instruments (Guttek) 9. Instrument sterilizer 10. Constructed carbon fiber electrodes 11. Nylon screws 2.4 mm length 12. Adhesive luting cement (C&B-Metabond^®^) 13. Orthodontic resin (ortho-jet crystal) 14. N-butyl 2 cyanoacrylate glue 15. Synthetic surgical suture (Nylon 3-0) 16. Dental drill 17. Saline solution (0.9%) 18. Hydrogen peroxide 19. Ophthalmic drops (artificial tears) 20. Antibiotic ointment (nitrofurazone 0.2 g) 21. Painkiller solution for injection (meloxicam 15 mg/ml) 22. Local injectable anesthetic (lidocaine 2%) 23. Syringes of 1 and 3 ml 24. Cotton swabs 25. Gauze 26. IceW3510

**TABLE 2:** Medication and dosage.

∙ Meloxicam ° Intramuscular injection 0.3 ml/kg ∙ Lidocaine ° Dilute to 0.5% for subcutaneous or intraincisional injection ° Inject less than 7 mg/kg ∙ Isoflurane ° 5% for induction ° 1–3% for maintenance during surgeryW8918

### Animals

Twelve adults (male *n* = 6) and (female *n* = 6) Wistar rats (*Rattus norvegicus albinus*) were obtained on postnatal day 21 (P21) from the vivarium of the Institute of Neurobiology in Queretaro, Mexico. Animals were individually housed in standard cages in a room with a 12:12 dark cycle/light, controlled temperature (23°C), and had free access to food. No rats had to be excluded due to complications with the model, treatment, or surgery.

### Experimental outline

**FIGURE 1:**
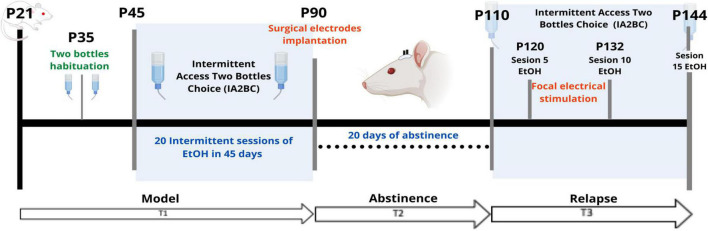
Experimental outline. T1 (Time 1) corresponds to the time encompassing the onset of IA2BC. T2 (Time 2) separates the 20-day period of abstinence. T3 (Time 3) corresponds to the phase of the model where the relapse phenomenon was observed. P, Postnatal age in days. EtOH, 20% ethanol.

The objective of this work was to validate the use of carbon fiber monopolar electrodes for chronic implantation in an ethanol self-administration model with longitudinal structural and functional MRI acquisition (Figure 1). Electrode implantation was performed once the *ethanol self-administration model* was established. For this model, we used the *Intermittent access two-bottle choice (IA2BC)* (Wise, 1973; Simms et al., 2008; Carnicella et al., 2014). Rats were individually housed at P35 and received at least 1 week of acclimatization with two bottles of water, the same to be used for the IA2BC model and handling. At P45, rats received 24-h sessions of free access to two bottle choices of water and 20% ethanol solution on Monday, Wednesday, and Friday, with 48-h withdrawal periods during the weekends. The placement of the bottles was alternated each drinking session to control for side preferences. During the withdrawal periods, rats received two bottles of water.

As for the timeline, the IA2BC model started on P45 (Time 1 or T1) and lasted for 45 days which included 20 sessions in total, ending in P90. The electrode implantation surgery for stimulation was done at P90, with 10 days of recovery from surgery (Time 2 or T2). Later, at P110, the rats were MRI-scanned (Time 3 or T3). Between P110 and P144 the IA2BC was reestablished with 15 sessions in total to measure relapse and alcohol use. It was during this time that the repeated focal electrical stimulation intervention was applied. T3 was subdivided into 3-time points: pre-stimulation (PreStim), stimulation (Stim), and post-stimulation (PoStim). Before stimulation, we randomly divided the sample into sham (placebo) (*n* = 6) and active (*n* = 6) stimulation groups. The sham stimulation group was treated exactly the same way as the active group, except that, during the stimulation sessions, they did not receive any stimulation.

### Carbon electrodes construction

The construction of the monopolar carbon electrodes was based on the Gallino et al. (2019) design. The electrodes were constructed using a cortical fiber of 0.28 mm in diameter and 1 cm in length (Easy Composites, Stroke on Trent, UK #CFROD-028) and an extracranial fiber of 2 mm in diameter and 5 mm in length (Good Winds, Mount Vernon, WA USA #CS070048) a 3D model of the electrode is shown in Supplementary Figure 1. The cortical fiber was isolated with 3 layers of spray rubber (Plasti Dip^®^) and both of the fibers were joined with a carbon epoxy (Atom Adhesives, Fort Lauderdale, FL, USA, #AA-CARB61), which conducts electricity between fibers. The final diameter of the cortical fiber was an average of 0.64 mm (Supplementary Figure 2). Finally, the resistance of the electrodes was measured with a voltmeter and marked with a range of 2–8 kω.

### Surgical carbon electrodes implantation

**FIGURE 2:**
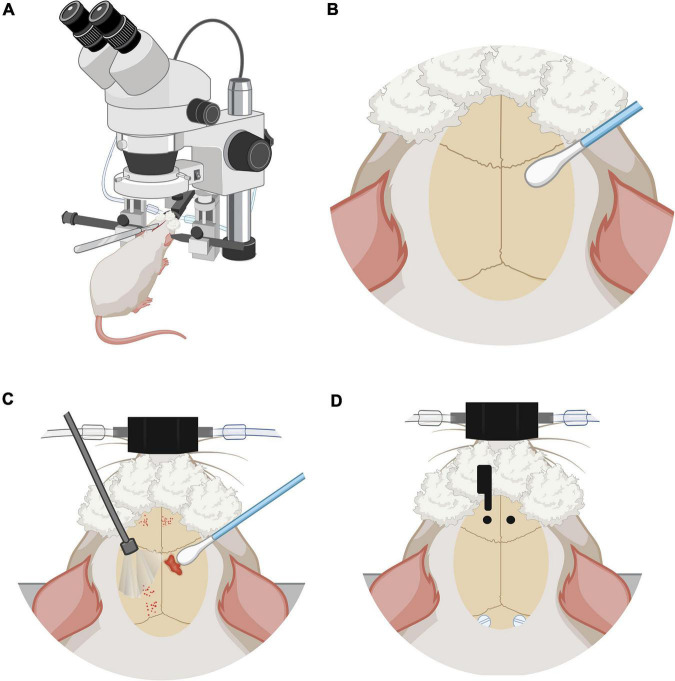
Anesthesia and stereotaxic surgery. **(A)** Rat placed in the stereotactic device. Rat under anesthesia vaporized with 3% isoflurane, on stereotaxic frame base with head fixation and a heated cushion under the body. **(B)** Preparation of the skull and control of local bleeding. Scalp incision on the midline following the direction of the mid-sagittal suture. **(C)** Curettage of skull aponeurosis and cleaning with swabs. **(D)** Coordinate marking of implantation coordinates of nylon screws and carbon electrodes under the microscope, directed by stereotaxic frame tower. Trepans on the skull are the entry site for the cortical implantation of the electrodes. Also visible are the nylon screws that are placed to keep the fibers fixed to the skull and maintain their chronicity.

From P80 to P90 rats were handled once a day for 5 min by caressing the top of their heads, where the electrodes will be placed later. This was to acclimate them to electrode manipulation and human-rat interactions. At P90 surgical procedure was performed; the technique was adapted from Matsumiya et al. (2012), Rigalli and Di Loreto (2016) and Santana-Chávez et al. (2020) (Figure 2).

#### Preparation for anesthesia (∼5–10 min)

Rats were anesthetized with vaporized isoflurane (∼5% induction) 50/50 isoflurane/oxygen mixture, administered in an induction chamber. A deep anesthesia state was verified with the absence of withdrawal reflexes to pain. The induction with isoflurane took between 5 and 10 min and was maintained during the positioning of the rat in the stereotaxic apparatus. The rat was placed in a prone position maintaining a permeable airway with the aid of the incisor immobilizer bar and the intra-aural position pencils. Artificial tears were then administered to the eyes and then covered with clean gauze. A rat gas anesthesia mask was used in the stereotaxic setup. The heart rate was maintained at ∼300 beats per minute and the respiratory thoracic movement was watched frequently and checked to be around 70 per min, and be regular and harmonic on both sides of the thorax. A heating pad placed on the stereotaxic apparatus base helped maintain the body temperature at 37°C during the surgery. Next, diluted lidocaine to 0.5% was applied subcutaneously at < 7 mg/kg, at the zone where the first incision would be made. The rats were injected with intramuscular meloxicam at 0.3 ml/kg to reduce intraoperative inflammation.

#### Stereotaxic procedure (∼1 h 30 min)

Once in deep anesthesia, the maintenance dose was modified from an average of 1–1.5%. A midline scalp incision of approximately 2 cm was made using a #20 scalpel blade (Figure 2A). The incision starts posterior to the line of the eyes. Bregma and lambda bony landmarks were exposed. Then the skin was moved to the sides with a self-retaining retractor. A peristome was used to separate the periosteum from the cranium bone. Next, hydrogen peroxide was used to achieve hemostasis with the help of cotton swabs. The importance of this step was to make sure that there was no bleeding and to dry the bone as much as possible (Figure 2B). With the scalpel blade, superficial cuts on the cranium bone were drawn and later washed with saline solution and hydrogen peroxide. Once the cranium was dry and there was no apparent bleeding, one drop of N-butyl 2-cyanoacrylate glue was applied to the exposed surface (Figure 2C).

#### Electrodes placement

Using the tip of the tower of the stereotaxic frame, electrodes were placed with Micropore breathable paper tape. Lambda and bregma bony landmarks were measured with the help of the tip of the electrodes and made the adjustment of ≤ 0.1 mm of a difference between both bony marks. Parallel to lambda, about half a centimeter from the midline, 2 mm diameter circles were marked to place plastic screws (P1tec, Roanoke VA, USA #0-80 × 3/32N). Subsequently, trepans were made to place the screws. Bleeding control was done with sterile 0.9% saline and cotton-tip applicators (Figure 2D). Finally, the position of the screws was sealed with adhesive luting cement (C&B-Metabond^®^).

#### Stereotaxic coordinates

Under the sight of a surgical microscope, a dental drill was used to make one-millimeter diameter holes bilaterally into the skull at the prelimbic cortex (PrL) (Paxinos and Watson, 2006) (Bregma 3.2 AP, 0.4 ML, 3.7 DV) or “area 32” (Paxinos and Watson, 2013). The electrodes were placed carefully and slowly, acquiring the DV coordinate 1 μm at a time. Once in place, they were sealed in position with adhesive luting cement, and a layer of resin was applied around and between all the arrays and screws to create a strong head cap fixed to the skull. Once the edges are smoothed and dried, simple stitches with nylon 3–0 closed the wound and left exposed the electrodes’ extracranial fiber.

#### Postoperative care

Before the rat was removed from the stereotaxic apparatus, the vaporized anesthesia was stopped, but the oxygen supply was kept until spontaneous movement appeared. Meanwhile, to manage pain relief, and inflammation, meloxicam (0.3 ml/kg) was intramuscularly injected. A nitrofurazone ointment was applied along the wound, and 1 ml of saline 0.9% solution was injected between shoulder blades to maintain the electrolyte balance. The eyes were cleaned and artificial tears were applied once again. When the rat awoke, it was returned to a clean cage, and the bottom of the cage was covered with paper towels to prevent choking or ingestion of the bedding. Each rat was housed individually in cages and monitored closely for 7–10 days after the surgery. If active bleeding was detected, reddened skin, or any signs of discomfort, the nitrofurazone ointment was applied up to 3 times a day. If not, one application per day for 3 days was enough to facilitate healthy scar formation. Four rats out of 12 required wound care three times a day for 3 days, after that the healing process progressed smoothly.

### Stimulation

**FIGURE 3:**
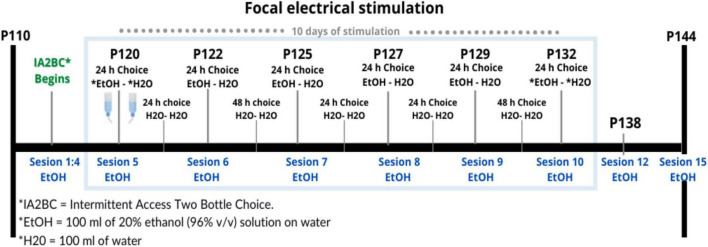
Timing of electrical stimulation and IA2BC during Time 3. Design of the schedule for repetitive focal electrical stimulation consisting of 10 days with a daily session of 10 min, this period is shown within the blue rectangle between days 120 and 132 of the age of the rat. In total there were 15 sessions where there was a bottle of water and a bottle of alcohol (EtOH-H_2_O) to choose to drink, shown in blue. Between each of these sessions, they were offered two bottles of water (H_2_O-H_2_O).

**FIGURE 4:**
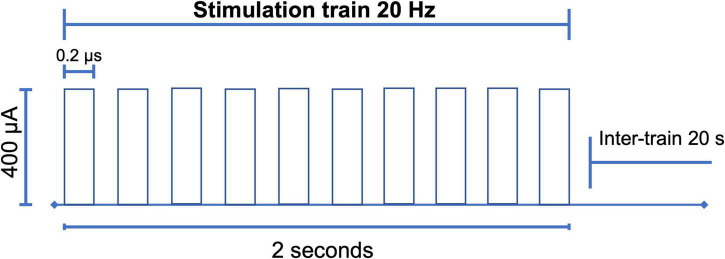
Electrical stimulation design. With the parameters of 300 pulses (duration = 0.2 ms, intensity = 400 μA) high frequency of 20 Hz, in trains of 10 pulses every 2 s with an inter-train interval of 20 s. After the choice of the carbon electrode, the research protocol was carried out for 10 consecutive days for 10 min (Levy et al., 2007; Gersner et al., 2010; Moshe et al., 2016).

Between P120 and P132, we began the stimulation protocol (Figure 3) during 10 sessions of 10 min for 10 consecutive days with 300 pulses (duration = 0.2 ms, intensity = 400 μA) at 20 Hz in 10 pulses per train of 2 s, and an inter-train interval of 20 s (Figure 4; Levy et al., 2007; Gersner et al., 2010). Stimulation was applied by means of GRASS S48 Square Pulse Stimulation connected to a GRASS stimulus isolation unit (SIU) and using metal alligators clips insulated exteriorly with Plasti Dip^®^ and welded to flexible electronic cable 20 AWG. Each rat remained in its individual housing and was habituated for 10 days, prior to treatment, to the connection with the alligator clips.

Just before being implanted intraoperatively, the electrodes were checked for conduction and verified with a voltmeter. Likewise, the length of the electrode was measured to determine that it preserved its integrity. As in the work of Olds and Milner (1954), the rats were in free movement, the stimulation region was related to the reward system, and given the intensity as well as the inter-train interval and the shape of the pulses, no visible locomotor impairment was expected, such as convulsions, tremors and gait deficiencies (Tehovnik, 1995). The above was qualitatively verified by the experimenter. None of the 12 rats reported in this experiment presented such side effects. It was reported that on average in the first 2 sessions of real stimulation, the 6 rats of the group showed interruption of exploratory, grooming, and sniffing behavior, remaining immobile for an average of 3 s aligned to the stimulation train. After that, only at the beginning of the stimulation session did they show this behavior throughout the rest of the sessions.

### Magnetic resonance imaging

MRI scanning was done at the Resonance Unit for Rodents and other Animals (URRA), Laboratorio Nacional de Imagenología por Resonancia Magnética (LANIREM) at the Instituto de Neurobiología, UNAM campus Juriquilla, located in Querétaro, Qro, Mexico. Before the acquisition, rats were injected subcutaneously with dexmedetomidine 0.012 mg/kg (Sirmpilatze et al., 2019). During imaging, rats were anesthetized with vaporized isoflurane 5% at induction and 0.5% in a 50/50 mixture of oxygen and vaporized anesthesia. Image acquisition was conducted using a 7T Bruker Pharmascan (Bruker Pharmasan 70/16, US) with a 2 × 2 surface coil and acquired using Paravision 6.0.1. A 3D FLASH sequence T2w with 2 repetitions TR = 30.76 ms, TE = 5 ms, flip angle = 10°, and FOV = 25.6 × 19.098 × 25.6 mm and an isometric voxel of 160 microns, and GE EPI sequences were: (1) TR = 1,000 ms, TE = 20 ms, flip angle = 60, slice thickness = 1 mm, FOV = 30 × 30, number of slices = 24, volumes = 600 and axial as primary slice orientation, (2) TR = 1,800 ms, TE = 20 ms, flip angle = 60, slice thickness = 0.75 mm FOV = 30 × 30, number of slices = 32, volumes = 334 and axial as primary slice orientation. Both sequences were performed at P110 to verify the location of the electrodes and at P144, at the end of the stimulation protocol. All images were converted from Bruker format to nifti using the brkraw tool v0.3.3 (Lee et al., 2020). Anatomical images were preprocessed using an in-house pipeline developed by Gabriel *A. Devenyi* based on MINC-toolkit-v2 and ANTs which performed the following steps: intensity normalization, center image, denoising, and registering in LSQ6 alignment.^1^ The preprocessing in this manuscript was done for better visualization. Functional images were preprocessed using RABIES software.^2^ A quality control (QC) of the acquisitions was performed based on considering spatial resolution and contrast, artifacts, and SNR.

## Results

**FIGURE 5:**
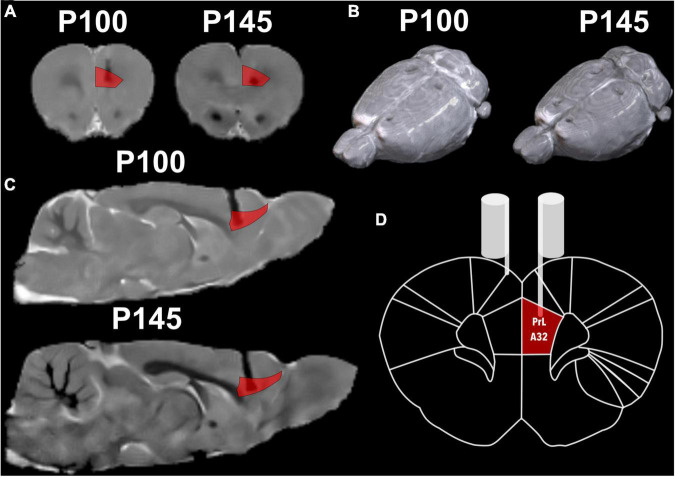
Structural MRI with a reference atlas (Paxinos and Watson, 2006) of Rat 2. **(A)** Coronal slide with atlas region PrL/A32 (prelimbic) region in red and the shadow of the electrode. **(B)** Render of rat brain dorsal view with electrodes marks and posterior to lambda two fixation screws. **(C)** Sagittal slide with atlas region PrL/A32 in red. **(D)** Schematic coronal slide from the atlas, with the left electrode on PrL/A32 and the right on the surface of the cortex.

Twelve rats conserved both stimulation electrodes and remained available for the stimulation protocol for the longitudinal follow-up. To ensure the feasibility of the electrode, we measured electrode resistance between sessions, and all the electrodes held their original values (2–8 kω). Additionally, MRI, as a non-invasive technique, was chosen for longitudinal monitoring, and the resulting images were anatomically compared to the Paxinos atlas (Paxinos and Watson, 2006; Figure 5). There were difficulties in adjusting the 2 × 2 surface coil in the male rats’ heads, hence to their body size plus the tip of the extracranial portion of the electrode, with this the acquisition resulted in a non-orthogonal position that later required an adjustment.

**FIGURE 6:**
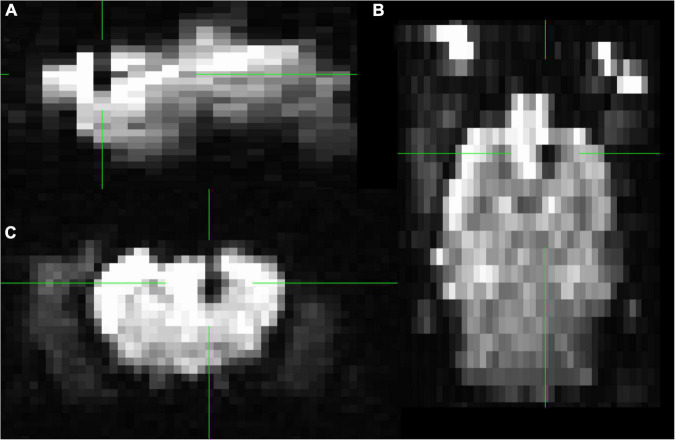
Functional MRI of rat 2. In the middle of the green markers, the black shadow of the artifact induced by the carbon fiber is seen, without affecting the areas surrounding the electrode. Slices **(A)** sagittal **(B)** axial **(C)** coronal. Group GE EPI sagittal view is shown in Supplementary Figure 3.

No 3D FLASH and GE-EPI acquisition of any rat at P100 and P145 were discarded after QC. On the GE-EPI sequence, the electrode is shown as a black area, and there was no geometric distortion around the implantation (Figure 6).

**FIGURE 7:**
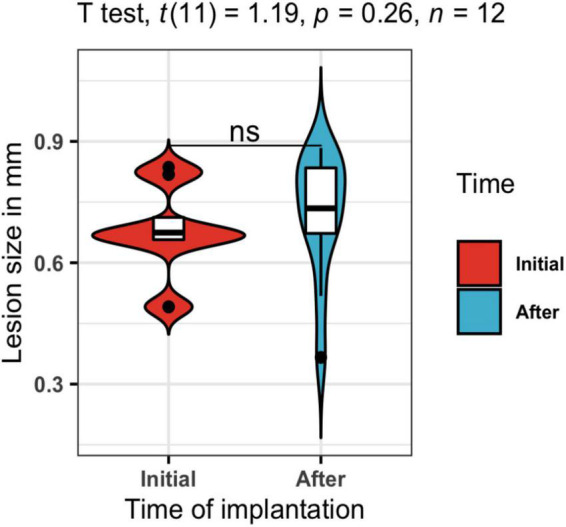
Lesion size in mm. A *t*-test was calculated to compare the lesion size at 2 points right after the surgical implantation P100 in blue (Initial) and at the end of the stimulation protocol P145 in red (After). The average lesion size of the twelve rats was not significantly increased after the treatment (stimulation) application, *t*(11) = 1.19, *p* = 0.26, *d* = 0.344.

Regarding the susceptibility artifact of the electrode in the T2w, the contrast of the area where the intracranial portion of the electrode was manifested as a black area. Around the electrode area, there were no displacement artifacts, geometric distortion, or signal loss (Hargreaves et al., 2011; Supplementary Figure 4). One of our main objectives was to prove that our electrodes would cause only a small lesion in the stimulation area, and with minimal inflammation. In the longitudinal follow-up of a rat in Figure 5, there were no structural changes measured with the 3D FLASH sequence, the measure of the major diameter in the landing area of the tip of the electrode was performed in all rats to calculate the mean and standard deviation of the lesions sizes. To corroborate the previous, we measured the lesion size at P100 and P145 of all twelve rats. A paired *T*-test was calculated to compare the lesion size at 2 points right after the surgical implantation P100 (Initial) (mean = 0.677 mm, *SD* = 0.111 mm) and at the end of the stimulation protocol P145 (After) (mean = 0.721 mm, *SD* = 0.152 mm). The average lesion size of the 12 rats was not significantly increased after the treatment (stimulation) application, *t*(11) = 1.19 *p* = 0.26, *d* = 0.344 (Figure 7).

**FIGURE 8:**
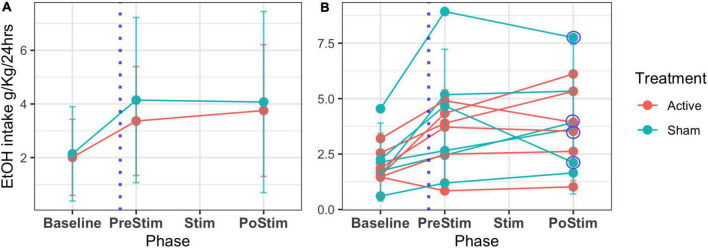
Mean EtOH intake and SD across phases of follow-up of the 12 rats. T1 or IA2BC development (baseline) is the 45 days where alcohol is offered in an elective model. The time for the surgery is marked with a blue dotted line (T2 or abstinence). T3 (relapse) comprehends a period before the stimulation treatment (PreStim) and the application of treatment under two conditions: sham/active (Stim) and a period of follow-up after the treatment (PostStim). **(A)** Mean and SD of the groups’ intake. **(B)** Individual mean ethanol intake and SD. Circled are the decreasing intakes between PreStim and PostStim evaluation of four rats.

Figure 8 shows the results on ethanol intake at all stages of the experiment (T1, T2, T3). The beginning of T3 is also the relapse phase, and all the rats increased their intake compared to the baseline. After the stimulation, there were no group differences in alcohol consumption. However, the individual plots showed high variability (Supplementary Table 1) in individual consumption. Two rats in the sham group, as well as 2 in the active group, reduced their ethanol consumption, while 2 rats in the active group and 1 in the sham group maintained their consumption stable. Two rats in the active group and 3 rats in the sham group increased their ethanol consumption after stimulation. Overall, our results show that 66% of the rats in the active group had a positive effect of stimulation/surgery, while 50% of the rats in the sham group also showed a reduction in consumption. A non-parametric method to compare means, Wilcoxon test, was used to assess the alcohol consumption of the sham and active stimulation groups at the times of pre-stimulation (PreStim) and post-stimulation (PoStim). For the active stimulation group, the median ethanol intake PreStim was 3.59 g/kg/24 h (IQR = 3), and PoStim 3.73 g/kg/24 h (IQR = 3.34) the differences were not significant *p* = 0.97 *r* = 0.0687 (Supplementary Figure 5A). The median ethanol intake PreStim for the sham stimulation group was 3.77 g/kg/24 h (IQR = 3.86), whereas the median PoStim was 3.27 g/kg/24 h (IQR = 3.40). The Wilcoxon test showed that the difference was not significant *p* = 0.77, with effect size *r* = 0.0395 (Supplementary Figure 5B).

## Discussion

Without a doubt it was challenging to assemble electrodes that met the requirements (Geddes and Roeder, 2003), of being: (1) MRI compatible, (2) able to perform focal electrical stimulation, and (3) well accepted by the body chronically. For future studies, we intend to demonstrate that they are capable of recording electrophysiological signals as LFPs, based on the report that they maintained their integrity throughout our study. Here we describe a method for the construction of carbon monopolar electrodes (Gallino et al., 2019) and propose their use for focal electrical stimulation as an intervention in a preclinical model of AUD (McBride and Li, 1998). Because of the study design, treatment follow-up did not allow us to draw a subsample to cohort and assess electrode viability, so it was monitored by electrical resistance and MRI. From the above we obtained that the rats that received active stimulation expressed immobilization behavior on average during the first 2 sessions, interrupting their exploratory, grooming, and sniffing behavior for an average of 3 s aligned with the stimulation train, similar to what was previously reported as behavioral evidence of the effect of electrical stimulation (Olds and Milner, 1954). The importance of the intended intervention was to stimulate a brain region that is thought to be the human homolog of the dorsolateral prefrontal cortex, which is the most commonly stimulated target region in rTMS. This region, the prelimbic cortex (PrL) or area 32, is an essential hub of the mesocorticolimbic network (Koob, 2013). The nucleus accumbens (NAc) and prefrontal cortex (PFC) PrL, or medial PFC in rats (Laubach et al., 2018) are brain regions that promise to be therapeutic targets for SUDs. Previously, implanted stimulation methods in PFC have decreased alcohol consumption in humans (Voges et al., 2013) and cocaine in rats (Levy et al., 2007). In this work, we stimulated rats that were previously exposed to IA2BC, and we found that four subjects with the greatest consumption of ethanol, decreased their intake in comparison to their consumption at the beginning of relapse or T3. To explain why both sham and active conditions on those four rats had an effect, we agree with Chakravarty et al. (2016) findings, who proposed that there were changes at the level of synaptic connections in both active and sham stimulation. In their study, active stimulation revealed a remodeling of the structure of the vasculature so that the diameter of the vessels increased. They also found increased volume in the subjects who received stimulation in remote regions, suggesting that neuroanatomical rearrangement also occurs in remote regions connected across multiple synapses (Stone et al., 2011). For future studies, we will analyze the functional and structural effects of stimulation in this AUD model (Bashir et al., 2011), and apply it to other SUD models.

Lastly, the model and the stimulation protocol have limitations. Firstly the sample acquisition time is 124 days, secondly the IA2BC is performed in conditions of reverse cycle (dark/light) for that a room that meets this criteria is required and thirdly, as previously described by Carnicella et al. (2014), only 30–40% of the rats in the IA2BC model become high drinkers. All of the above conditions interfere with the sample size, and it is due to this that possibly no differences were established, taking into account the tendencies to decrease the consumption of the subjects in the groups with higher alcohol. Nevertheless, we are working on further experiments to increase the sample size.

In summary, this work describes chronic, MRI-compatible, carbon electrode implantation, and the use of focal electrical stimulation on a preclinical model of AUD with a longitudinal follow-up. Our findings suggest the possibility of decreasing ethanol intake after the stimulation protocol. Further work is needed to elucidate the effects of stimulation in AUD and other SUDs.

## Data availability statement

The raw data supporting the conclusions of this article will be made available by the authors, without undue reservation.

## Ethics statement

The animal study was reviewed and approved by Comité de Bioética del Instituto de Neurobiología UNAM Campus Juriquilla, México.

## Author contributions

AL-C, GR-P, and EG-V contributed to the conception and design of the study. AL-C performed the surgeries and creation of the electrodes, performed data analysis, visualizations, and wrote the first draft of the manuscript. AL-C and DA-V worked on the main ethanol model and acquired MRI data. AL-C and EG-V wrote sections of the manuscript. EG-V was in charge of the funding of the research. All authors contributed to manuscript revision, read, and approved the submitted version.

## Footnotes

1
https://github.com/psilantrolab/Documentation/wiki/Preprocessing-Rat-Structural-in~vivo

2
https://github.com/CoBrALab/RABIES
