# Supplementary material for: Focal electrical stimulation on an alcohol disorder model using magnetic resonance imaging-compatible chronic neural monopolar carbon fiber electrodes
Source: Front Neurosci. 2022 Sep 29;16:945594. doi: 10.3389/fnins.2022.945594 (PMC9558902; doi:10.3389/fnins.2022.945594)
Supplement: Supplementary file 1 [file Data_Sheet_1.PDF]

Supplementary figures and tables

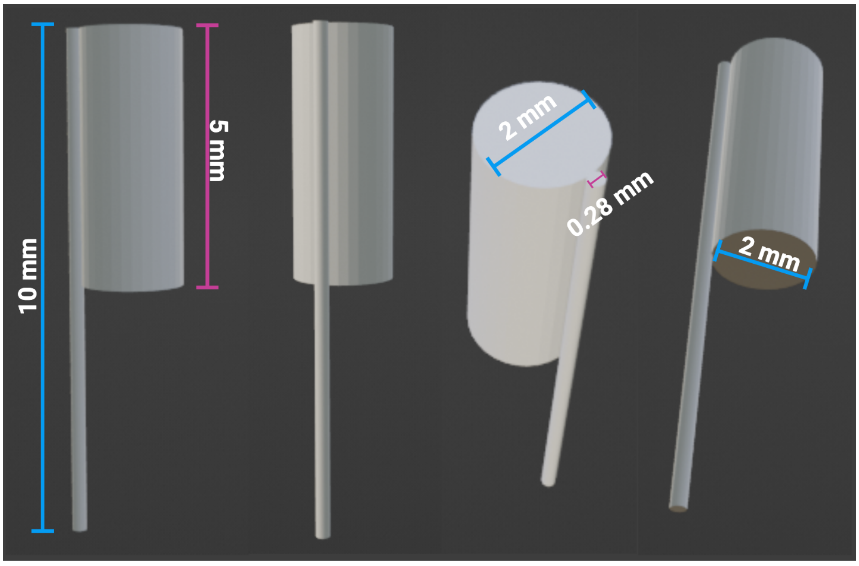

Figure S1. Electrode 3D design. 3D model of the carbon electrode consisting of a 1 cm long fiber with a diameter before the coating, of 0.28 mm, and a short extracranial fiber of 0.5 mm and 2 mm in diameter.

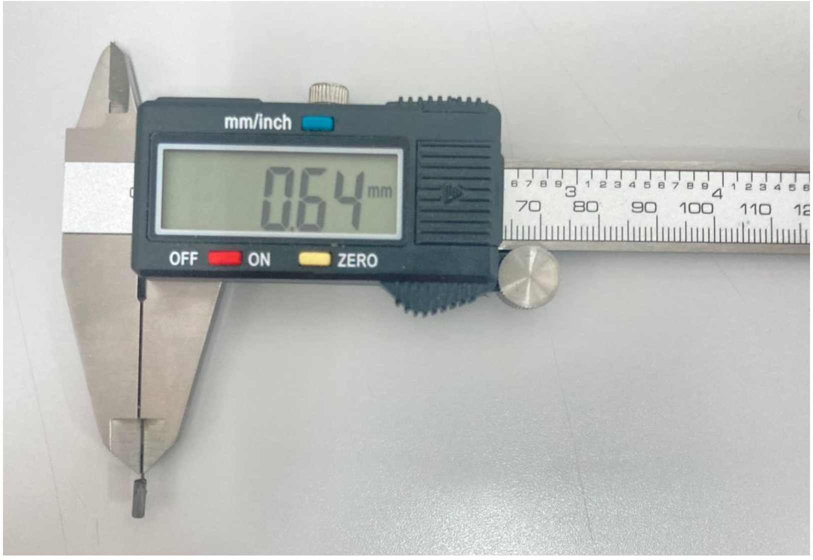

Figure S2. Electrode extracranial fiber diameter. 0.64 mm diameter of the cortical fiber after coating with three layers of PlastiDip.

Rat	Ethanol intake gr/kg/24 hrs		
	Mean ± Standard deviation	Max (Min)	Range
1	2.42 ± 2.34	11.51 (0.28)	11.23
2	2.02 ± 1.47	6.85 (0.18)	6.67
12	3.27 ± 2.64	10.08 (0.26)	9.82
16	3.42 ± 3.11	13.23 (0.00)	13.23
17	2.56 ± 1.66	6.38 (0.39)	5.99
18	2.48 ± 1.28	6.20 (0.39)	5.81
25	3.67 ± 1.84	8.16 (1.22)	6.94
26	6.07 ± 2.63	11.30 (1.67)	9.62
33	1.16 ± 1.14	5.26 (0.12)	5.15
39	0.87 ± 0.81	3.37 (0.14)	3.23
45	3.25 ± 1.80	9.21 (0.21)	9.00
48	2.83 ± 1.68	7.66 (0.63)	7.03

Supplementary Table 1. Ethanol intake gr/ kg / 24hrs of the twelve rats.

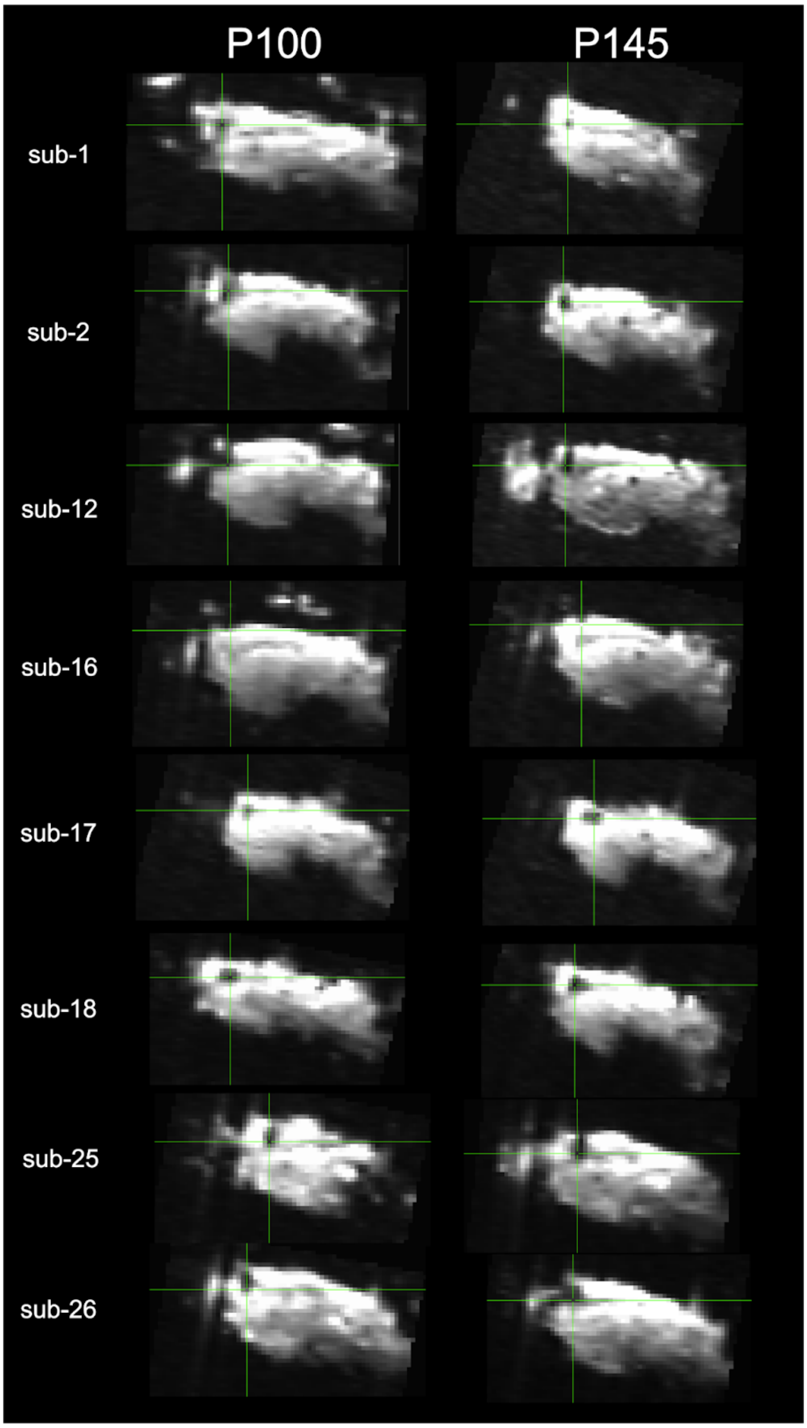

Figure S3. Sagittal view of GE EPI sequences for 8 of the 12 rats. A black shadow between the green markers shows the carbon electrode susceptibility artifact, limited to the electrode region.

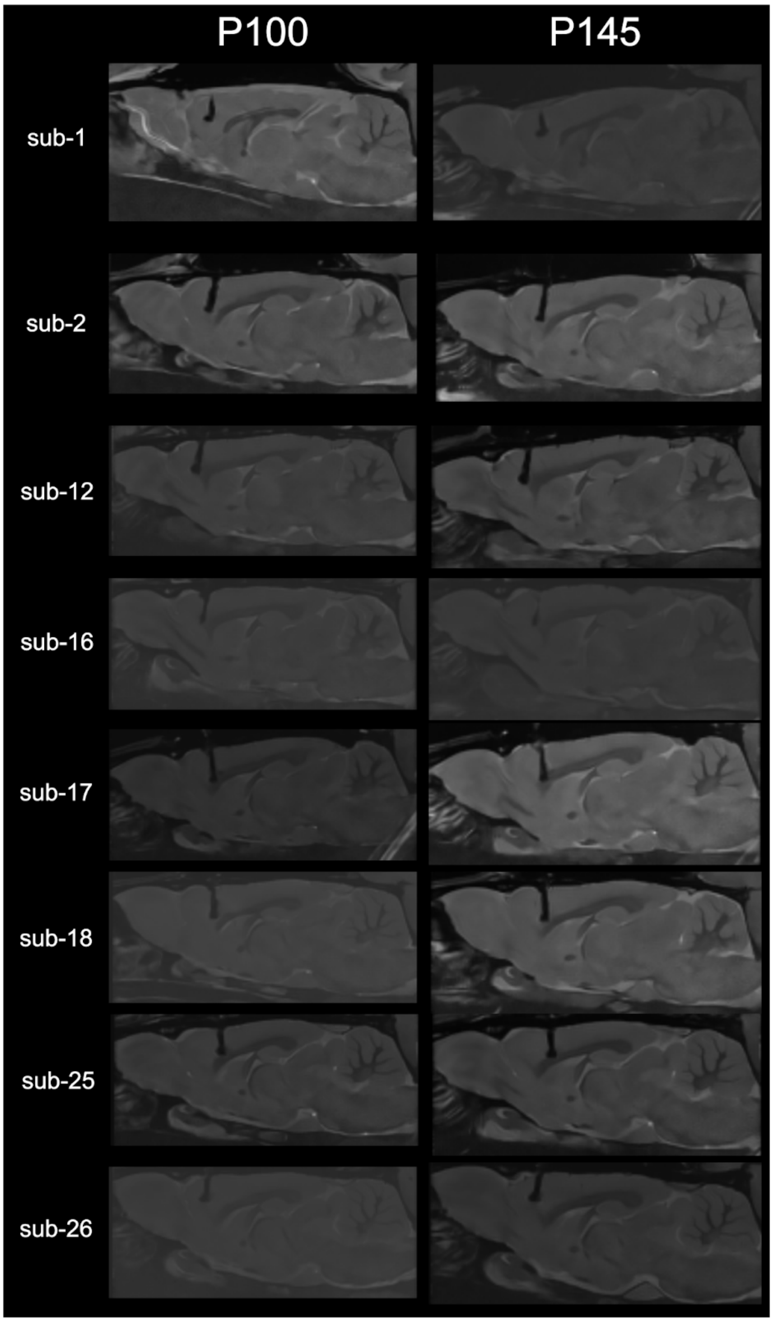

Figure S4. Sagittal view of 3D FLASH sequences for 8 of the 12 rats. A black shadow shows the artifact created by the cortical carbon fiber, with no apparent distortion, loss of geometry, or loss of signal in the areas surrounding the electrode.

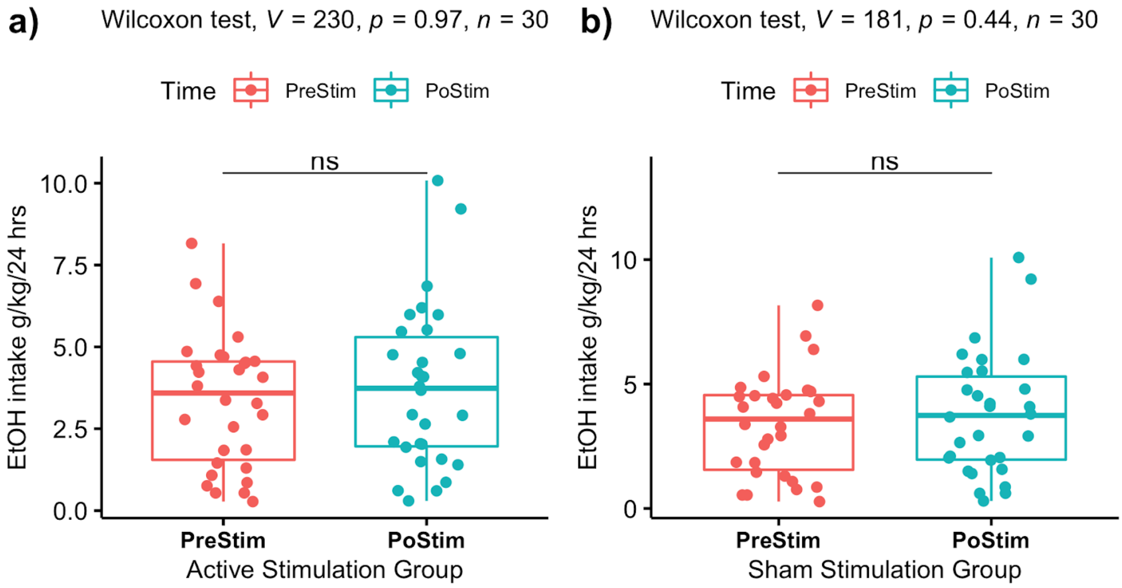

Figure S5. Ethanol main intake g/kg/24 hrs for the active stimulation and sham stimulation group. A Wilcoxon test was calculated to compare the alcohol consumption of the sham and active stimulation groups at the times prestimulation (PreStim) in red and poststimulation (PoStim) in blue. a) For the active stimulation group the median ethanol intake PreStim was 3.59 g/kg/24 hrs (IQR = 3), and PoStim 3.73 g/kg/24 hrs (IQR = 3.34) the differences were not significant p = 0.97 r = 0.0687. b) The median ethanol intake PreStim for the sham stimulation group was 3.77 g/kg/24 hrs (IQR = 3.86), whereas the median PoStim was 3.27 g/kg/24 hrs (IQR = 3.40). The Wilcoxon test showed that the difference was not significant p = 0.77, effect size r = 0.0395.
